# Monitoring and Analysis of COVID-19 Pandemic: The Need for an Empirical Approach

**DOI:** 10.3389/fpubh.2021.633123

**Published:** 2021-07-08

**Authors:** Martí Català, Miquel Marchena, David Conesa, Pablo Palacios, Tomas Urdiales, Sergio Alonso, Enrique Alvarez-Lacalle, Daniel Lopez, Pere-Joan Cardona, Clara Prats

**Affiliations:** ^1^Comparative Medicine and Bioimage Centre of Catalonia, Fundació Institut d'Investigació en Ciències de la Salut Germans Trias i Pujol, Badalona, Spain; ^2^Department of Physics, Universitat Politècnica de Catalunya (UPC-BarcelonaTech), Barcelona, Spain; ^3^Experimental Tuberculosis Unit, Fundació Institut d'Investigació en Ciències de la Salut Germans Trias i Pujol, Universitat Autònoma de Barcelona, Badalona, Spain; ^4^Centro de Investigación Biomédica en Red de Enfermedades Respiratorias, Madrid, Spain

**Keywords:** COVID-19, risk indexes, risk diagram, epidemic monitoring, COVID-19 outbreak

## Abstract

The current worldwide pandemic produced by coronavirus disease 2019 (COVID-19) has changed the paradigm of mathematical epidemiology due to the high number of unknowns of this new disease. Thus, the empirical approach has emerged as a robust tool to analyze the actual situation carried by the countries and also allows us to predict the incoming scenarios. In this paper, we propose three empirical indexes to estimate the state of the pandemic. These indexes quantify both the propagation and the number of estimated cases, allowing us to accurately determine the real risk of a country. We have calculated these indexes' evolution for several European countries. Risk diagrams are introduced as a tool to visualize the evolution of a country and evaluate its current risk as a function of the number of contagious individuals and the empiric reproduction number. Risk diagrams at the regional level are useful to observe heterogeneity on COVID-19 penetration and spreading in some countries, which is essential during deconfinement processes. During the pandemic, there have been significant differences seen in countries reporting case criterion and detection capacity. Therefore, we have introduced estimations about the real number of infectious cases that allows us to have a broader view and to better estimate the risk. These diagrams and indexes have been successfully used for the monitoring of European countries and regions during the COVID-19 pandemic.

## Introduction

The current pandemic produced by coronavirus disease 2019 (COVID-19) is strongly impacting the world. With more than 150 million confirmed cases and more than 3 million reported deaths, the pandemic has been a worldwide tragedy, with consequences impacting far beyond these numbers. In addition to the health disaster in all the countries in the world, the control measures had important consequences, not only the expected socioeconomic derivatives but also emotional (1, 2), educational (3, 4), or cultural (5, 6) consequences, to cite but a few. Therefore, this emergency situation has required constant monitoring at multiple levels—from the city neighborhood tracking of local outbreaks to a global continental perspective for socioeconomical decisions coordinated at the interstate level. Different political actors need different pieces of information to take decisions regarding mobility, schools, or the redirection of health resources, among others.

Unfortunately, the spreading dynamics of the severe acute respiratory syndrome coronavirus 2 (SARS-CoV-2) is largely unknown and certainly not sufficiently characterized to develop mechanistic models that properly predict its propagation in the medium term. For example, there is uncertainty in the literature regarding the influence of temperature and humidity in its transmission, with reports indicating both a very small (7) and a relatively large (8) effect. The apparent clustering behavior (9) of the transmission adds an important layer of uncertainty regarding under which conditions the virus propagates optimally. This renders complicated mechanistic models of propagation useless in the sense of providing useful quantitative information. Relevant indicators for policy makers must come from empirical epidemiological models of the cumulative cases and fatalities of the pandemic.

One of these indicators is the effective reproduction number, R_t_, that is normally assessed by means of an SIR model (i.e., a compartment model based on Susceptible-Infectious-Recovered flows) (10) or likelihood-based estimation procedures based on the generation time interval method (11). These kinds of models require previous parametrization. In this sense, we propose a more transparent and empiric way to characterize the spreading of the epidemic that we call ρ_t_. This index measures the ratio between new cases at an interval of 5 days. It is thus a parametrization-free parameter that we will show is closely related to R_t_. When combined with the evaluation of active cases, it provides an empirical quantification of the epidemiological risk in a given region.

The manuscript is structured as follows. First, the *Methods* describe the empirical indexes used on daily tracking of epidemics (12). Then, the *Results* section shows that they are good short-term predictors, allowing a proper evaluation of the state of the epidemic.

## Methods

The reported data from government sources about the pandemic are large and normally not unified in their criteria. They must be properly assessed and curated to obtain useful and truthful information, especially about the trend of its spreading. Our main aim is to analyze whether the situation is improving or getting worse, so that it can be used by policy makers when deciding different socioeconomic measures. To address this issue, we have developed or adapted three indexes to compare different situations and evaluate the resurgence risk: an empiric estimation of the reproduction number, an index of the contagious pool and a risk index of the effective potential growth. Moreover, and looking for an effective and proper communication of the epidemic situation in a certain country, we have built a discrete scale that assesses the level of incident cases. These indexes are adapted to COVID-19 but can also be used in any pandemic or epidemic.

### Empiric Indexes

#### Empiric Reproduction Number

Classically, epidemiology uses the effective reproduction number (*R*_*t*_) (9) to measure the velocity at which the epidemic is propagated during an outbreak. It is a measure of the mean number of new infections caused by an infectious individual. Let *R*_0_ be the value of *R*_*t*_ before the epidemic starts, that is, at *t* = *t*_0_. To compute these parameters, SIR and SEIR (susceptible-exposed-infectious-removed) models (13) are traditionally used. However, these models are difficult to address COVID-19 pandemic due to the high number of unknowns about inherent parameters (14). In addition, classical SEIR models are driven by susceptible population availability, while the evolution of this pandemic is mainly governed by the control measures like confinement or social conscience regarding the hygiene rather than by susceptible population.

Other methods to calculate *R*_*t*_ are also available based on the estimation of the generation time between two correlated infections and the probability of infection along the disease of an individual (11). The lack of complete knowledge of such factors at the beginning of the epidemic suggested to assume a naiver description.

We propose an empiric definition of the propagation rate (*ρ*_t_), which is defined as the number of newly infected in the last 3 days divided by the number of newly infected during 3 days τ days ago:

$$\begin{array}{l} \rho_{t}\left(t-1,\tau \right)=\frac{nc\left(t-2\right)+nc\left(t-1\right)+nc\left(t\right)}{nc\left(t-2-\tau \right)+nc\left(t-1-\tau \right)+nc\left(t-\tau \right)} \end{array}$$

where *nc*(*t*) is the number of new cases at time *t*, and *τ* is the incubation period, which in COVID-19 case is estimated to be around 5 days (15, 16). Furthermore, 5 days also correspond to the average generation time (time between generations) (17), giving rise to a simple first-order approximation to the effective reproduction number R_t_. Then, similarly to the use of R_t_, if *ρ*_*t*_ > 1, the epidemic is growing because the number of new cases today is bigger than the number of new cases 5 days ago. Otherwise, the incidence of new cases is lower and the epidemic is reducing. When *ρ*_*t*_ = 1, the epidemic is not growing nor reducing and the new number of cases is maintained.

Propagation rate is very sensitive to noise effects. Thus, at initial and final stages, when the number of new cases is small, the behavior of *ρ*_*t*_ does not represent the reality. Besides, the temporal evolution of *ρ*_*t*_ is subject to human reporting data effects, such as the weekend effect (12). To address these issues, we define *ρ*_*T*_ as the moving average of ρ_*t*_ for *T* days:

$$\begin{array}{l} \rho_{T}\left(t,\tau \right)=\frac{1}{T}\overset{\frac{T-1}{2}}{\underset{i=-\frac{T-1}{2}}{\sum }}\rho_{t}\left(t+i,\tau \right). \end{array}$$

In the following, we will set *T* = 7 days in order to avoid the weekend effect (18). Note that this definition is only valid for odd values of *T*. Otherwise, one would find non-integer values of *t*, which is the time variable in days and must be an integer.

#### The 14-Day Attack Rate: A Measure of Contagious People

Parameters *ρ*_*t*_ and *ρ*_7_ are useful to identify if an epidemic is growing; however, it is not the same to obtain a *ρ*_*t*_ bigger than 1 with a large amount of potentially contagious individuals or, on the contrary, if the fraction of potentially contagious individuals is small. The number of contagious individuals is a difficult quantity to estimate since contagious people are not necessarily detected. An index commonly used to follow active cases in COVID-19 is the 14-day attack rate (i.e., new cases of last 14 days per 10^5^ inhabitants, *A*_14_) (19, 20), which is defined as follows:

$$\begin{array}{l} A_{14}\left(t\right)=\frac{N\left(t\right)-N\left(t-14\right)}{population}\cdot 10^{5}, \end{array}$$

where *N* is the number of cumulative reported cases in a country, and *population* is the population of the country or region under consideration.

Nevertheless, the reported cases criterion is very different through the countries due to many facts: type of test reported, reporting frequency, update of reported data temporal series, number of available tests, percentage of diagnosed cases, biased subpopulations that are over/underdiagnosed, etc. (21). Thus, the number of reported cases is not as representative as one would expect. As for the reported deaths, there is also variability among countries but at lower levels (22). Then, it is possible to calculate the diagnosis rate (*DR*) from these data, which allows us for the estimation of the real number of cases (*N*_*est*_) (22). This estimation agrees with different seroprevalence testing done afterward (23). We can define the estimated 14-day attack rate (*A*_14,*EST*_) as:

$$\begin{array}{l} A_{14,EST}\left(t\right)=\frac{N_{EST}\left(t\right)-N_{EST}\left(t-14\right)}{Population}\cdot 10^{5}. \end{array}$$

Assuming a constant diagnosis rate, this equation can be simplified:

$$\begin{array}{l} A_{14,EST}\left(t\right)=\frac{A_{14}\left(t\right)}{DR\left(t\right)}. \end{array}$$

By symmetry, we will name the 14-day attack rate evaluated with reported data as *A*_14,*REP*_.

#### Effective Potential Growth

The effective potential growth (EPG) is a risk index that evaluates the potential epidemic growth at short term. It is defined as the product between the mean propagation rate of the last 7 days (*ρ*_7_), which reflects the velocity at which the epidemic is spreading, and the 14-day attack rate (*A*_14,*REP*_), which accounts for the contagious population that could propagate at that rate:

$$\begin{array}{l} EPG_{REP}\left(t\right)=\rho_{7}\left(t\right)\cdot A_{14,REP}\left(t\right). \end{array}$$

In fact, this product provides, under constant conditions, an order of magnitude of the expected number of new cases that will be diagnosed (i.e., that will be reported) for the next 14 days per 10^5^ inhabitants. However, *EPG*_*REP*_ is a magnitude that changes over time, so it can be used for evaluating the risk associated with this potential growth at any moment during the epidemic. This index was used to decide which Catalan Sanitary Regions were deconfined, among other criteria.

If we want to evaluate the risk based on the estimated pool of contagious population, *A*_14,*EST*_, we obtain the expression:

$$\begin{array}{l} EPG_{EST}\left(t\right)=\frac{EPG_{REP}\left(t\right)}{DR\left(t\right)}. \end{array}$$

### The Biocom-Cov Scale

Popular language often uses sea-related vocabulary to describe the dynamics of COVID-19 in a region or country: first wave to refer to the first peak, secondary waves to describe subsequent outbreaks, or tsunami to refer to a totally uncontrolled outbreak, among others. The Douglas Scale is a discrete way of classifying the situation of the sea that considers, among others, the height of the waves. We propose a discrete way of classifying the situation with regard to daily new cases in what we name Biocom-Cov scale.

Looking at orders of magnitude, 200 active cases per 100,000 inhabitants pose an impossible challenge, while 20 active cases can be dealt with by public health officials if they are properly found and the structure of test and trace is in place. Assuming that active cases are well-represented by A_14_, corresponding average daily new cases would be 200/14 = 14.3 daily new cases. Then, the 14 daily new cases are placed as the threshold for the highest level. Here, 100 active cases per 100,000 inhabitants are a highly problematic situation from the control perspective. This situates another important threshold at 7–8 daily new cases per 100,000 inhabitants. Similarly, five daily new cases per 100,000 should count as rather high situation, and two daily cases (around 30 active cases) should be the limit of moderate. With these ideas in mind, we build the scale shown in Table 1, which gives a complete and accurate picture of the situation.

**Table 1:** Biocom-Cov scale to assess the epidemic degree of a region.

**Pandemic degree**	**Daily new incident cases per 10^**5**^ inhabitants**
0	0
1	0–0.1
2	0.1–0.5
3	0.5–1.25
4	1.25–2
5	2–3
6	3–5
7	5–8
8	8–14
9	>14

## Results

### ρ_t_, an Indicator of Contagious Velocity

**Figure 1:**
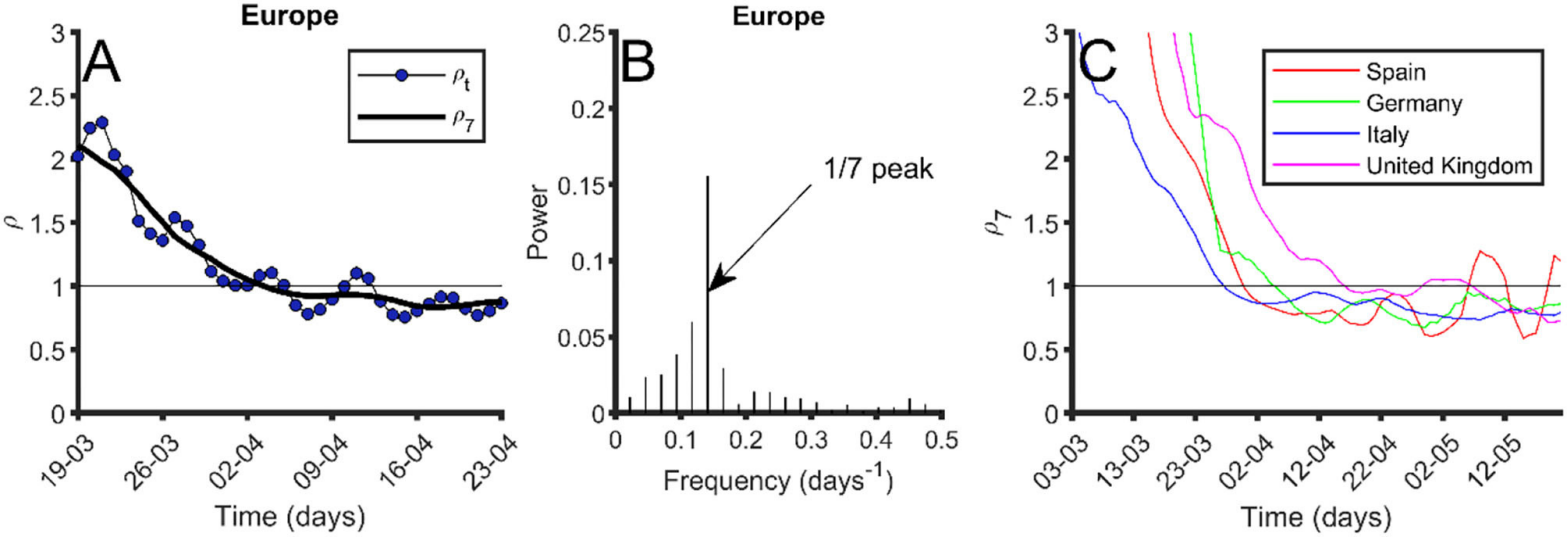
Index *ρ*_*t*_evolution and periodicity. **(A)** In blue dots, evolution of *ρ*_*t*_ in Europe; black thick line, 7-day moving average of *ρ*_*t*_ (*ρ*_7_). **(B)** Fourier transform of *ρ*_*t*_. The peak at frequency 1/7 days^−1^ is pointed out. **(C)** Seven-day moving average of *ρ*_*t*_ (*ρ*_7_) for different European countries: Spain (red), Germany (green), Italy (blue), and United Kingdom (purple). Data were obtained from the European Center for Disease Prevention and Control (24) on May 25, 2020.

The evolution of the empiric reproduction number, *ρ*_*t*_, is crucial to evaluate the dynamics of the pandemic. The number of new cases increases until *ρ*_*t*_ gets smaller than 1. A stable *ρ*_*t*_ below 1 is needed to reduce the new number of cases. The blue dots in Figure 1A show the weekend effect in *ρ*_*t*_ at the European level caused by a weekend delay in data recording. Therefore, oscillations with a 7-day periodicity are observed. Figure 1B shows the Fourier transform of *ρ*_*t*_, where the 7-day oscillation period is clearly identified. Therefore, the 7-day moving average in *ρ*_*t*_ is necessary to study the epidemic dynamics, given by *ρ*_7_. Figure 1A shows the comparison between daily *ρ*_*t*_ and the averaged *ρ*_7_. The comparison of *ρ*_7_ through various countries allows for the exploration of differences in COVID-19's dynamics among countries. Figure 1C shows the evolution of *ρ*_7_ in Germany, Italy, Spain, and United Kingdom. Italy was the first European country where the pandemic started, while the fastest pandemic control (i.e., decrease in *ρ*_7_) was in Germany. Spain followed a control dynamics similar to the one in Italy, even slightly faster. Italy, Germany, and Spain have managed to stabilize *ρ*_7_ below 1. Nevertheless, *ρ*_7_ is still sensitive to fluctuations in data, which is the reason why it increases above 1 in two short periods for Spain. This country experienced some problems with data reporting the last weeks, and a couple of spikes in new data masks the real decreasing global trend. United Kingdom shows a slower control of the epidemic, with a *ρ*_7_ that remained around 1 for several weeks, and that finally dropped below 1.

### Reported and Estimated Risk Indexes

Risk indexes *ρ*_7_, A_14_, and EPG (on their reported and estimated versions) have been used in the daily tracking of the epidemic in European and several non-European countries. Index *ρ*_7_ provides a quantification of the velocity at which the epidemic is being spread; the higher, the worse. Index A_14_ provides a way to quantify active cases, i.e., it is an indicator of the contagious people that can spread the virus at the velocity *ρ*_7_. Finally, EPG evaluates the risk that results from both indexes. A high *ρ*_7_ with a very low A_14_ does not represent high risk, and this is reflected by low values of EPG. We consider two types of EPG, EPG_REP_ and EPG_EST_ evaluated, respectively, with the data reported by countries and with the estimated real incidence.

**Figure 2:**
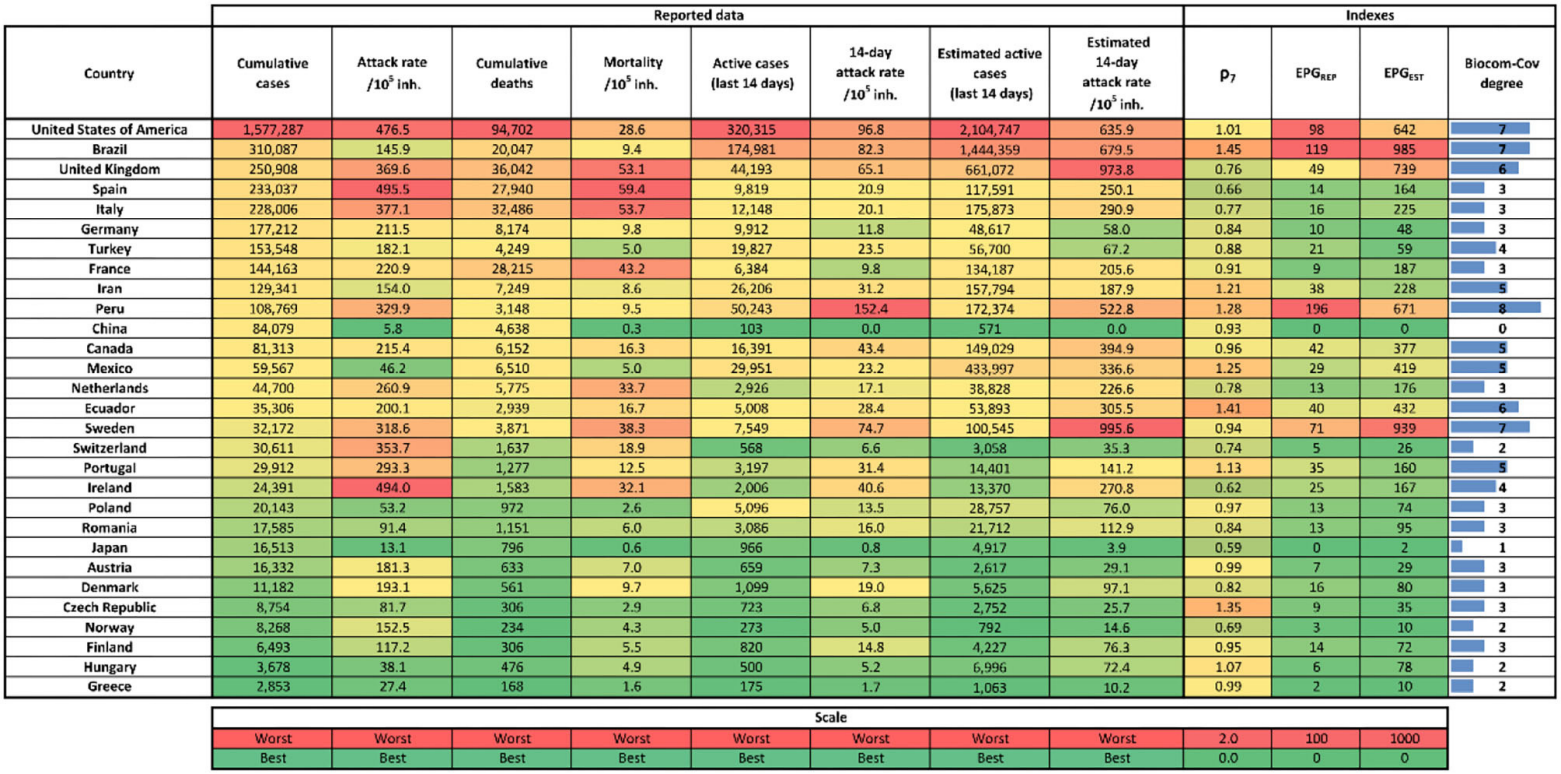
Table with last indexes and reported cases value as of May 22, 2020. Left to right columns are: country, cumulative reported cases, number of total cases per 10^5^ inhabitants (attack rate), cumulative number of reported deaths, number deaths per 10^5^ inhabitants, reported number of new cases last 14 days (active cases), reported active cases per 10^5^ inhabitants (14-day attack rate), estimated number of new cases for the last 14 days (active cases), estimated active cases per 10^5^ inhabitants (14-day attack rate), 7-day moving average empiric reproduction number (*ρ*_7_), effective potential growth of reported cases (EPG_REP_), estimated effective potential growth (EPG_EST_), and Biocom-Cov degree. Each column has its own color scale as seen at the bottom of the figure. Data were obtained from the European Center for Disease Prevention and Control (24) and World Health Organization (25) on May 23, 2020.

Figure 2 shows the values of several variables and indexes (including *ρ*_7_, A_14,REP_, A_14,ST_, EPG_REP_, and EPG_EST_) for different countries on May 22, 2020. At that date, countries at the highest risk according to EPG_REP_ were Perú, Brazil, and USA. If we look at the risk given by EPG_EST_, the most worrying situation was that of Brazil followed by Sweden, UK, USA, and Perú. A comparison between the estimated and reported EPG is useful in determining which countries are underreporting.

Figure 2 also incorporates the Biocom-Cov degree of each country. As shown, this scale immediately facilitates a good visualization of the country situation beyond the need for other precise numerical indicators. In order to bypass the weekend effect, we assign the Biocom-Cov degree looking at the average of daily new cases in the last week.

**Figure 3:**
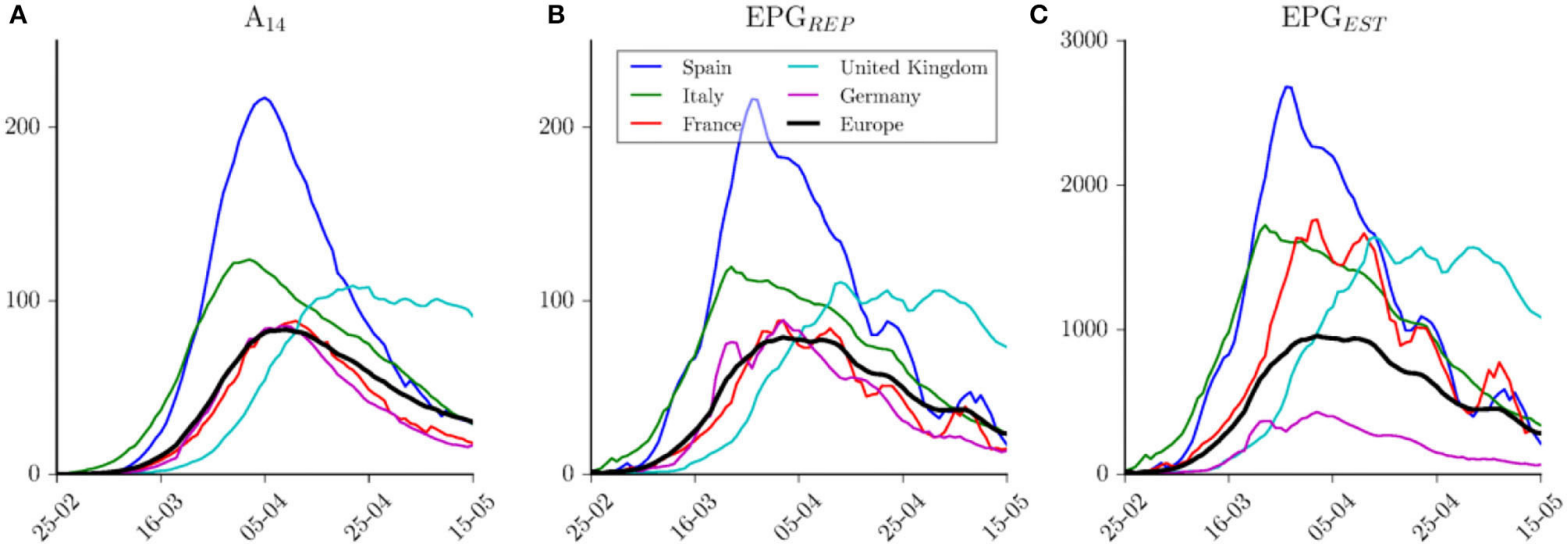
Indices evolution among time. **(A)** Evolution of reported 14-day attack rate per country. **(B)** Effective Potential Growth evolution per country computed with reported cases. **(C)** Effective Potential Growth evolution per country computed with estimated cases. Blue lines for Spain, green for Italy, red for France, cyan for United Kingdom, purple for Germany, and black for Europe. Data were obtained from the European Center for Disease Prevention and Control (24) on May 16, 2020.

Time evolutions of A_14,EST_, EPG_EST_ and EPG_EST_ are shown in Figure 3 for the five European countries with the highest number of total reported cases (UK, Spain, Italy, France, and Germany). Figure 3A shows that Italy was the first country in Europe to reach the peak of contagious cases. Spain is the country that arrived at the highest number of contagious people per 10^5^ inhabitants, nearly doubling that of Italy (217 vs. 124), which was the country with the second highest incidence. UK curve shows that this country has transited a plateau rather than a peak, thus illustrating the delay in controlling the epidemic.

Figure 3B shows the evolution of EPG_REP_ in these countries. Spain was the one that reached the highest risk on March 28, 2020, when it had an EPG_REP_ of 214. This index also provides similar risk levels achieved by the other four countries, which at the same time would be similar to the one reached by Europe as a whole. Moreover, if we compare the same countries using the estimated EPG_EST_ (Figure 3C), we observe that Germany has been in a better situation than the one reflected by reported data when compared with other countries. According to this index, Italy, UK, and France have had a similar level of estimated risk but at different moments, slightly higher (i.e., worse) than the one shown by European average. Risk reduction in Spain is faster than the one observed in Italy; in fact, they are in a similar level now. Germany and France show a similar EPG_REP_ but totally different estimated EPG_EST_. The number of reported deaths in this pair of countries is quite different, with a six-fold increase between them. This leads to the estimation of a higher diagnosis rate in Germany than the one in France (25 vs. 7%) (22). From this analysis, it is important to note that the time scale in the reduction of the number of active cases is larger than the time scale observed during the growing phase.

### Risk Diagrams: A Tool to Evaluate Risk

**Figure 4:**
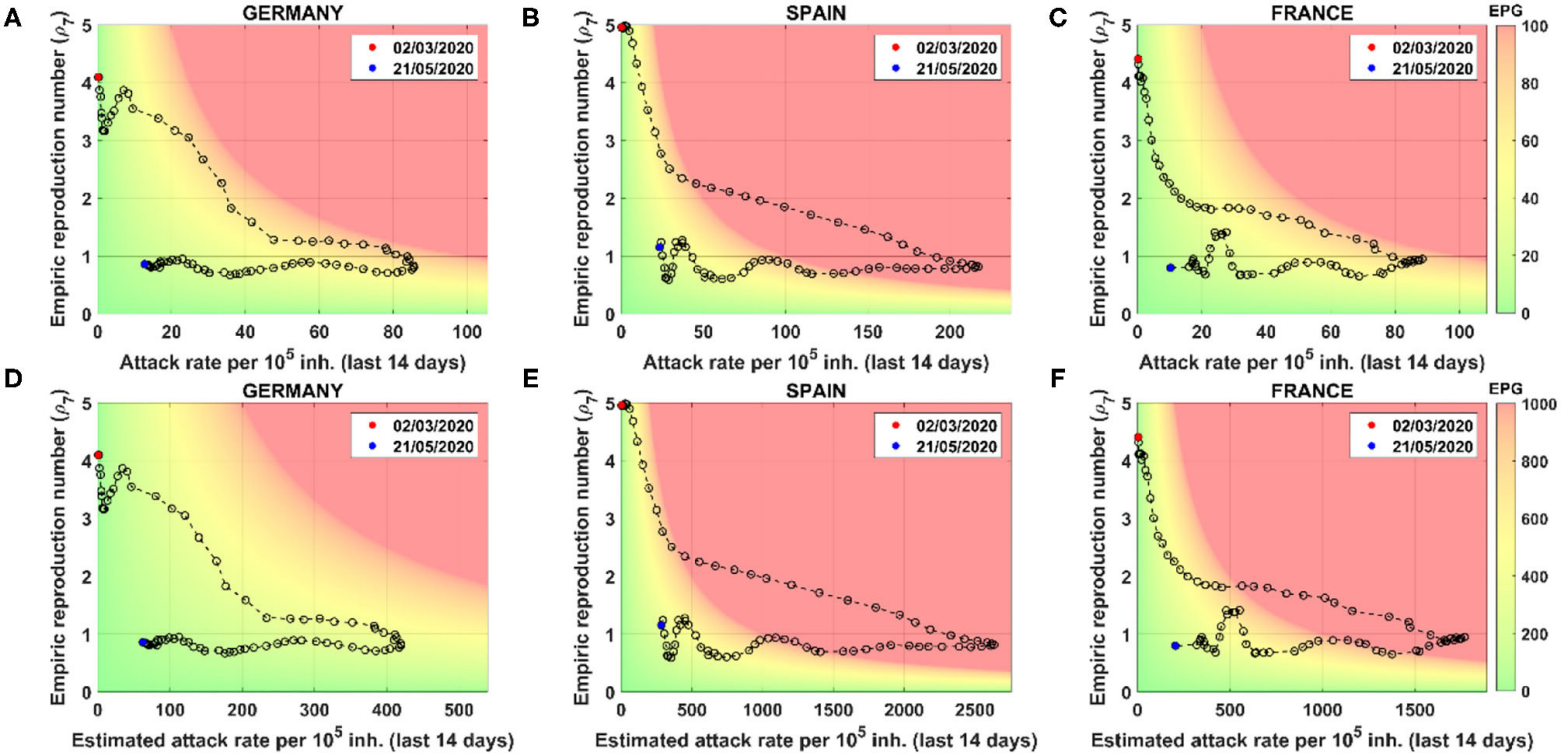
Risk diagrams for different countries using reported and estimated cases. Each point represents a different day. Starting and final days are marked by date. Color code depends on Effective Potential Growth (EPG). Two color codes are used: one for reported (top right bar) and another for estimated (bottom bar). Using reported cases: **(A)** Germany, **(B)** Spain, and **(C)** France risk diagrams. Using estimated cases: **(D)** Germany, **(E)** Spain, and **(F)** France risk diagrams. Note that x-scales are different, but plots can be compared through the color background.

The risk diagram is a tool to visualize the evolution of a region or country in terms of *ρ*_7_ (y-axis), A_14_ (x-axis), and EPG (background color) with either reported or estimated data. Figure 4 shows the risk diagrams for France, Spain, and Germany. In the upper part, risk diagrams using reported data (Figures 4A–C), in the bottom part, risk diagrams using estimated data (Figures 4D–F). We consider a risk situation (red) for EPG_REP_ > 100 and EPG_EST_ > 1,000.

The color code is related to the ability of a country or region to do contact tracing, setting at 1,000 estimated real cases the red as the threshold where it is impossible to carry it out. The maximum number of daily PCR tests per 10^5^ people performed sustainably has been of the order of 50–100 (26) in affected countries. At this level of testing, it would take between 10 and 20 days to process all active cases, which is precisely the time it takes for infected people to get seriously sick or die. Unless the infrastructure is scaled up dramatically in the future, 1,000 active cases are impossible to test and trace nowadays.

The general dynamics along the risk diagram is quite similar for all countries. At the beginning of the pandemic, the attack rate is low while the propagation velocity is high (*ρ*_7_ > 1). When restrictions and physical distancing measures are applied, the velocity of propagation drops down, but, since it is still above 1, the attack continues to increase. The inflection point is achieved when *ρ*_7_ crosses the threshold of 1. At that moment, the number of new infected cases starts to decrease; meanwhile, *ρ*_7_ remains below 1. Then, the curve moves toward the green zone.

Analyzing case-by-case, we see that Spain was the country that was in a worse situation since they went further in the risk zone, where there are more than 214 cases per 10^5^ inhabitants expected for the next 14 days. Looking at the estimated diagrams, we see that Germany was the country with a better estimated real situation, since they did not reach the danger zone.

**Figure 5:**
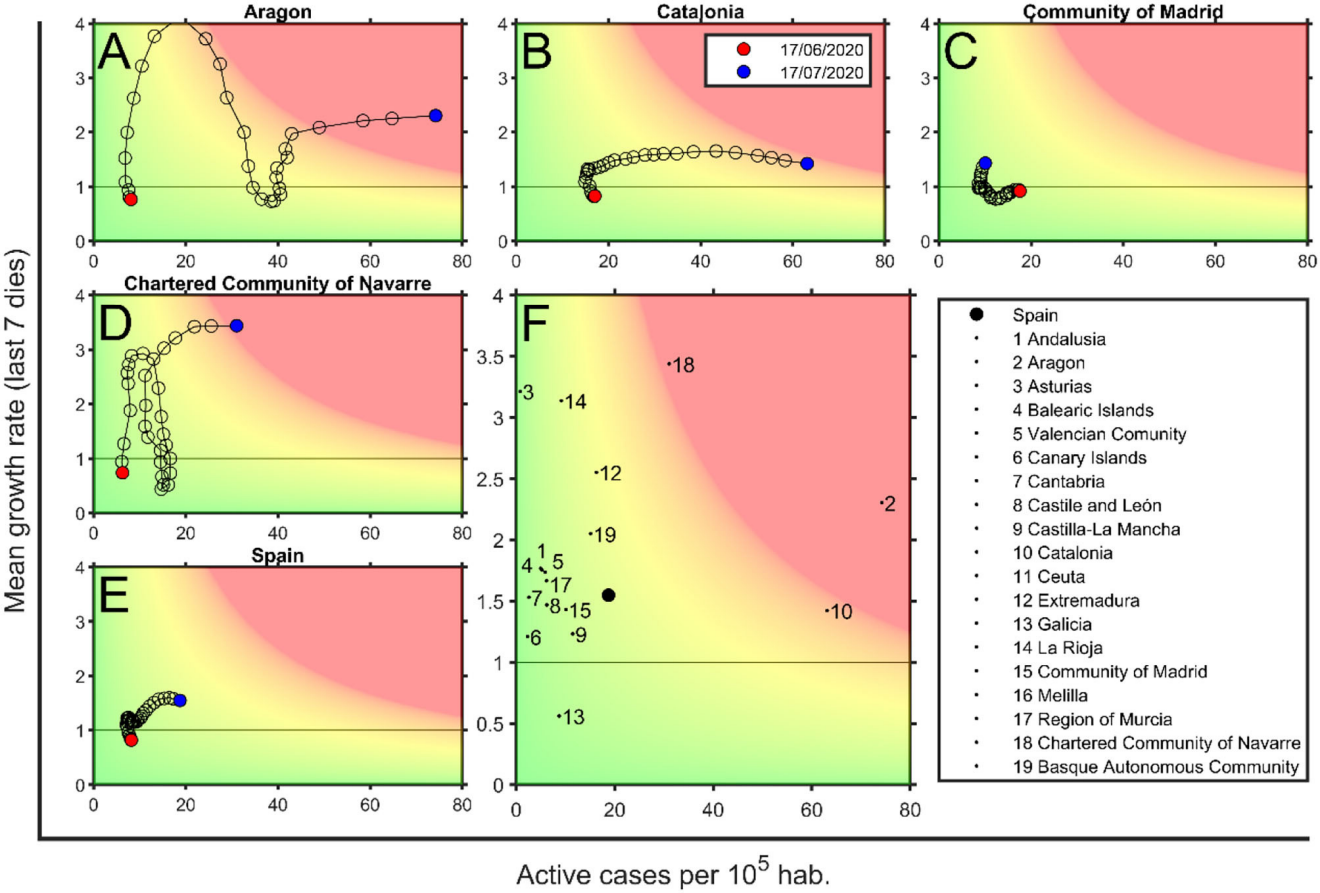
Risk diagrams for Spain and some of its regions on July 17, 2020. **(A)** Aragon, **(B)** Catalonia, **(C)** Community of Madrid, **(D)** Community of Navarra, and **(E)** Spain risk diagrams. Each circle corresponds to a day, last day is marked with a black filled circle. **(F)** Spain region situation, where each dot is the position for each Spanish region on July 17, 2020 (see legend), and filled black circle is the situation of Spain. Background color depends on the Effective Potential Growth (EPG) risk. Red marks EPG = 100 and green is for EPG = 0; there is a linear degradation between both. Zones with an EPG higher than 100 are also marked in red. Data are obtained from Datadista (27) and Instituto de Salud Carlos III (28) on July 18, 2020.

The analysis of a full country by using only this risk diagram could lead to a misleading visualization of the real situation. In these figures, we are studying the situation of these countries by considering them as a whole. However, the situation in the different regions of each country could be very different, and a deeper analysis must be done. The fragmentation of the country into several regions allows us to better understand the situation as well as how the propagation has occurred. This information is crucial for policymakers to properly develop novel strategies during confinement and deconfinement. For instance, the regional variability for the cases of Spain is shown in Figure 5. COVID-19 risk diagrams are updated daily at Català et al. (12).

### Risk Diagrams as a Tool to Detect Uncontrolled Outbreaks

Most European countries have overcome an initial peak and then entered a long tail that is expected to finish when herd immunity is achieved or an efficient vaccine is available. Meanwhile, the main concern of regions and countries is the early detection of outbreaks and their evaluation from the risk perspective, so that physical distancing measures or mobility restrictions can be imposed, if necessary. The main threshold between control and uncontrol is the presence of community contagions, i.e., the loss of contagious chain traceability.

**Figure 6:**
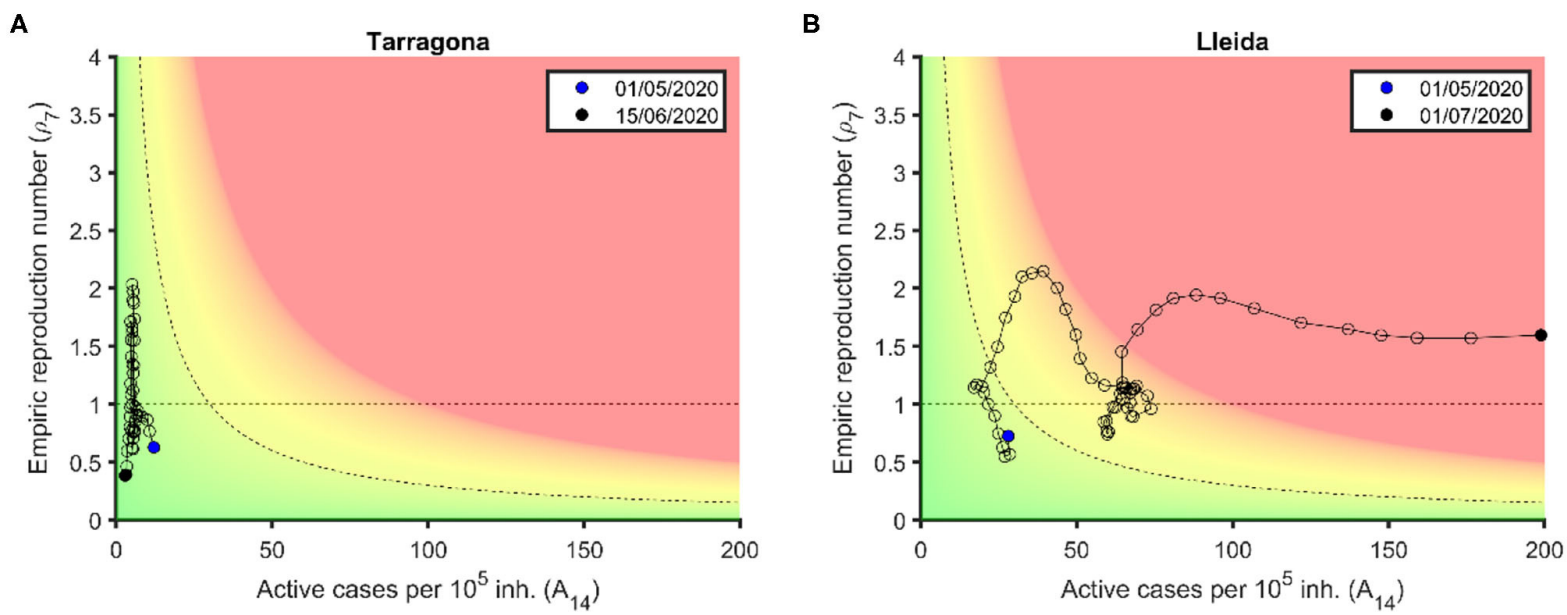
Controlled and uncontrolled outbreaks in the risk. **(A)** Tarragona province: Two consecutive increases in *ρ*_7_ occur in the green zone and can be controlled with simply test and trace. **(B)** Lleida province: An initial increase in *ρ*_7_ occurs in the green-yellow zone and can be controlled through a slowing down of de-escalation process, while a second increase in *ρ*_7_ enters the red zone and becomes an uncontrolled outbreak. Data are obtained from Datadista (27) and Instituto de Salud Carlos III (28) on October 23, 2020.

In risk diagrams, the loss of traceability is represented by a red zone, where control by test and trace is not possible. Figure 6 shows the usefulness of risk diagrams to distinguish local controlled outbreaks from uncontrolled ones. In this figure, we show controlled and uncontrolled outbreaks that start with an initial increase in *ρ*_7_ up to 2. First, we can observe two outbreaks in the control zone as a certain increase in *ρ*_7_ that is not followed by an increase in active cases (Tarragona province). Therefore, this is observed as a simple up–down trajectory of the plot in the low risk zone. Contrarily, an outbreak that is not well-delimited and immediately controlled drags the curve to the right (Lleida). While this dynamics occurs in the yellow zones, control with soft measures is possible (in this case, a slowing down of the de-escalation process). When a new increase in new cases leads the curve toward the red zone, it strongly suggests the presence of community transmission and the need for restrictions in mobility or social interactions.

## Discussion

We have shown that *ρ*_7_ and A_14_ are good indicators for assessing epidemiological risk in regions or countries. They can be replaced by alternative ways of measuring spreading rate and contagious potential, but an indicator for each one must be considered if the risk is to be evaluated properly. In particular, the proposed way to assess *ρ*_7_ is very sensitive to changes in the transmission dynamics, which can be especially useful to detect those changes. A slight change in this methodology, applying the 7-day moving average to the cases (*nc*) instead of the *ρ*_7_, provides a more stable evaluation of the transmission rate, which is less affected by artifacts such as changes in the diagnosis protocols or underreporting of holidays.

We have proposed the EPG index as a simple way to account for both factors. During the growth phase (pre-peak) of the epidemic, EPG is used to track the dynamics of the epidemic, and when it increases above a threshold, EPG can indicate the need for new control measures. Nevertheless, we consider the main focus of this index to be the management of the deconfinement process. It is essential that de-escalation phases take into account the relative epidemiological risk of the region or country in the context of the health system robustness and operability, together with the capability to incorporate contact tracing strategies that avoid new outbreaks. In addition, an increase in EPG also can be used as an alarm symptom when looking for secondary outbreaks.

Risk diagrams are a good way to visualize the situation and dynamics of countries in this sense. Its color scale is based on EPG values and its relation with the ability to trace given by the testing infrastructure typical in European countries. This scale, however, can be particularly adapted to each country, considering the level of incidence that the country can assume with the local ability to test and trace. Finally, those countries with a diagnostic level below 10% should try to incorporate estimations on the management of the epidemic (for instance, using EPG_EST_ instead of EPG_REP_).

Most important limitations of any empirical approach to the COVID-19 pandemic are related to the diagnosis effort and bias (22). An insufficient diagnostic effort may affect not only an underreporting of cases but also an underreporting of deaths. In addition, holiday periods that modify the basic structure of 5 working days plus 2 holidays per week can generate artifacts on the observed data. In any case, and in conclusion, the use of empirical indexes like EPG and the risk diagrams can help with the monitoring of the COVID-19 epidemic and to address relevant questions, for example, the classification analysis of the evolution of the cases or the appearance of new outbreaks. In particular, any change in historical trends due to the appearance of a more transmissible variant or to the increase in the vaccination coverage among a certain population can be easily detected using such an empiric approach, since it lacks a mechanistic hypothesis to be revised.

## Data Availability Statement

The original contributions presented in the study are included in the article/supplementary material, further inquiries can be directed to the corresponding author.

## Author Contributions

CP, P-JC, and DL conceived the study. MC, MM, DC, PP, and TU wrote the codes and prepared tables and figures. MC, MM, SA, EA-L, DL, P-JC, and CP analyzed the results, discussed the implications, and proposed reformulations of the indexes. MC, MM, PP, SA, EA-L, and CP prepared the draft version. All authors read and approved the final version.

## Conflict of Interest

The authors declare that the research was conducted in the absence of any commercial or financial relationships that could be construed as a potential conflict of interest.
